# Relationship of maternal obesity and vitamin D concentrations with fetal growth in early pregnancy

**DOI:** 10.1007/s00394-021-02695-w

**Published:** 2021-10-17

**Authors:** Qianqian Zhang, Chen Zhang, Yi Wang, Jiuru Zhao, Haiyuan Li, Qianwen Shen, Xiaoli Wang, Meng Ni, Fengxiu Ouyang, Angela Vinturache, Hao Chen, Zhiwei Liu

**Affiliations:** 1 Departments of Neonatology, School of Medicine, International Peace Maternity and Child Health Hospital of China Welfare Institution, Shanghai Jiao Tong University, 910 Hengshan Road, Shanghai, 200030 China; 2 Departments of Neonatology, School of Medicine, Children’s Hospital of Shanghai, Shanghai Jiao Tong University, Shanghai, 200040 China; 3 Shanghai Key Laboratory of Embryo Original Diseases, Shanghai, 20030 China; 4 Ministry of Education and Shanghai Key Laboratory of Children’s Environmental Health, School of Medicine, Xinhua Hospital, Shanghai Jiao Tong University, Shanghai, 200092 China; 5 Department of Obstetrics and Gynecology, Queen Elizabeth II Hospital, Alberta, Canada

**Keywords:** Vitamin D, Obesity in pregnancy, Crown-rump length, Early fetal growth restriction

## Abstract

**Purpose:**

To evaluate the effects of the association between first trimester vitamin D (VitD) concentrations and increased prepregnancy body mass index (BMI) on early fetal growth restriction (FGR).

**Methods:**

This retrospective cohort study included 15,651 women with singleton pregnancy who delivered at the International Peace Maternity and Child Health Hospital between January 2015 and November 2016. Women were classified in two groups based on their serum 25(OH)D vitamin levels status: VitD sufficient (SUFF) group and VitD insufficient or deficient (INSUFF/DEF). The cut-off point for VitD concentration was 50.00 nmol/L. Comparisons were made between women with normal prepregnancy body weight (BMI 18.5–23.9 kg/m^2^) and overweight and obese (OWO) women (BMI > 24.0 kg/m^2^). Early FGR was defined as first-trimester gestational age-adjusted crown-rump length (CRL) in the lowest 20th centile of the population. Multivariate logistic regression was used to evaluate the association between maternal serum 25(OH)D levels and prepregnancy BMI with first trimester CRL and early FGR.

**Results:**

In VitD INSUFF/DEF group, the first trimester CRL was decreased (*P* = 0.005), and the risk of early FGR was increased by 13% (95% CI 1.04–1.24, *P* = 0.004) compared to the VitD SUFF group. In OWO group, the first trimester CRL was also significantly decreased (*P* < 0.0001), and the risk of early FGR was significantly increased by 58% (95% CI 1.40–1.78, *P* < 0.001) compared with normal weight group. Furthermore, there was a significant combined effect of maternal VitD concentrations and OWO on CRL (*P* for interaction = 0.02) and the risk of early FGR (*P* for interaction = 0.07).

**Conclusion:**

Sufficient first trimester serum 25(OH)D concentration was a protective factor for early fetal growth, especially among OWO mothers. Chinese Clinical Trial Registry (Registration number: ChiCTR1900027447 with date of registration on November 13, 2019-retrospectively registered).

**Supplementary Information:**

The online version contains supplementary material available at 10.1007/s00394-021-02695-w.

## Introduction

Maternal vitamin D (VitD) deficiency is a worldwide public health problem, with an estimated prevalence ranging from 18 to 84% among pregnant women [1]. There is an association between VitD deficiency during pregnancy and an increased risk of preeclampsia [2], gestational diabetes [3, 4], primary caesarean section [5], preterm birth [6] and low birthweight [7]. However, the evidence on the association between VitD levels and pregnancy outcomes is conflicting. A recent overview of systematic reviews on the effectiveness of VitD supplementation on perinatal outcomes showed no significant benefit from VitD in terms of preeclampsia, gestational diabetes, preterm birth, stillbirth, low birthweight, and cesarean section [8]. In contrast, a Cochrane review published a year prior found that supplementing pregnant women with VitD alone may potentially reduce the risk of preeclampsia, gestational diabetes, low birthweight, and postpartum hemorrhage while having little or no effect on the risk of preterm birth [9, 10]. An association between maternal VitD levels and fetal bone development have been demonstrated [11, 12]. VitD deficiency associates not only with reduced infant birth size [4], but also with other adverse offspring health consequences such as rickets, skeletal problems, type 1 diabetes, schizophrenia, and asthma [13].

Among the risk factors for VitD deficiency, maternal obesity is a notable one. Prepregnancy obesity predicts poor vitamin D status in mothers and their neonates [14], probably related to sequestering of VitD_3_ in the adipose tissue with decrease in VitD bioavailability [15, 16]. Overweight and obesity have become increasingly common among women of childbearing age in both developing and developed countries. In the USA, 55.8% of women of reproductive age are overweight or obese (OWO) [17], whereas in China, OWO prevalence was reported at 24.8% among young women and adolescent girls [18]. Thus, it is conceivable to assume that a large population of women of reproductive age is VitD deficient.

In addition to VitD’s essential role in calcium homeostasis and bone metabolism [19], recent evidence shows that VitD also plays a fundamental role in early pregnancy, in the process of conception, implantation and development of the placenta itself [20, 21], as a regulator of trophoblast invasion in early pregnancy, crucial event for fetal growth and development. Most of the clinical and epidemiological studies on VitD in pregnancy focus mainly on the associations with fetal bone development [7, 22, 23]. Evidence is accruing to show that deficiency in VitD, even less profound, may lead to suboptimal bone size and density from early pregnancy [24]. However, information on the consequences of maternal VitD insufficiency and early fetal growth restriction (FGR) is limited.

First-trimester crown-rump length (CRL) measured by ultrasonography is mainly used to estimate gestational age based on the assumption there is no growth variation at this time [25]. Discrepancies between last menstrual period and ultrasound estimations cannot only be a consequence of the gestational age adjustment [26] but can also be the result of differences in embryonic growth [27]. The first-trimester gestational age-adjusted CRL can be used as an indicator of early FGR [28].

To this end, the aim of this study was to assess the relationship between maternal VitD circulating levels and early fetal growth, in obese and normal weight women.

## Materials and methods

### Study participants

In this retrospective cohort study, women with singleton pregnancy who underwent prenatal assessment and delivered at the International Peace Maternity and Child Health Hospital (IPMCH) in Shanghai, China from January 2015 to November 2016 were eligible for inclusion. Maternal prepregnancy weight and height were collected on a standard questionnaire at the first antenatal visit interview. VitD levels were routinely measured in the first trimester of pregnancy in all eligible participants. Women who had VitD concentrations assessed after 13 weeks of gestation or had a diagnosis of diabetes and thyroid disease before pregnancy were excluded because these disorders are known to associate with FGR [29–31]. Women with uncertain date of last menstrual period or irregular menstrual cycle (menstrual cycle length outside of range 28 ± 4 days) were also excluded from the study.

**Fig. 1:**
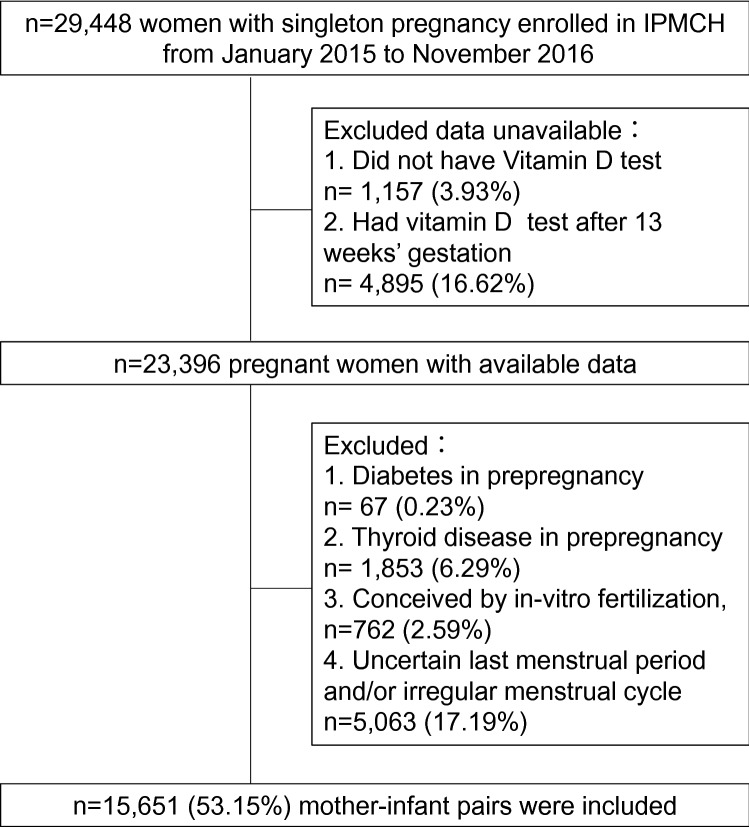
Flowchart of the participants included in the analysis. *IPMCH* International Peace Maternity and Child Health Hospital

Clinical obstetric and neonatal information was retrieved from the medical records database. As illustrated in the flowchart (Fig. 1), from 29,448 women with singleton pregnancy enrolled in the study, 1157 women without VitD testing, 4895 women with VitD testing after 13 weeks (second trimester), 67 women with history of diabetes, 1853 women with history of thyroid disease, 762 women who conceived by in-vitro fertilization, and 5063 women with an uncertain last menstrual period or irregular menstrual cycle were excluded from the study. Ultimately, 15,651 mother-infant pairs were included in this study.

The above-mentioned medical conditions (i.e., diabetes, thyroid disease) were diagnosed according to clinical protocols and defined using the International Classification of Diseases, 10th Revision, Clinical Modification.

### VitD testing and classification

Circulating serum concentrations of 25(OH)D were used in this study to evaluate the VitD status of the participants. Briefly, maternal 5 mL blood samples collected from the median cubital vein at the first antenatal visit (9–13 weeks of gestation) were centrifuged to obtain the serum within 2 h from collection. The serum was stored at room temperature until 25(OH)D measurements, that occurred within 6 h from the specimen storage. Quantitative analysis of 25(OH)D was performed using chemiluminescence microparticle immunoassay in an Architect I2000SR automatic analyzer (Abbott Diagnostics, Chicago, USA). Serum 25(OH)D concentrations were calculated based on a standard curve, following standard clinical procedures performed by qualified lab technicians. The detection range of the analyzer was between 2.00 and 400.00 nmol/L. The intra- and inter-assay coefficients of variation were < 5%. To date, there is no consensus on the optimal level of VitD for pregnancy health, and a reference serum level during pregnancy has not been determined [32]. Thus, this remains an area of active research. As such, the VitD cut off levels in this study have been chosen based on previous published studies in pregnant women [33]. VitD levels were used as categorical variable in this study. Women with serum 25(OH)D levels > 50.0 nmol/L, were considered VitD sufficient (SUFF), and women with levels < 50.0 nmol/L were considered as insufficient or deficient (INSUFF/DEF).

### Fetal ultrasonography

In our hospital, first trimester ultrasound (< 13 weeks) is part of the routine antenatal care. All participants underwent real-time transabdominal ultrasonography with a full bladder. CRL was measured to the nearest millimeter in a sagittal plane with the fetal head in a neutral position, and the maximum length from the cranium to the caudal rump was recorded [34]. Medical doctors certified in first trimester ultrasound performed the ultrasonic scans and measured the CRL using a 5–7 MHz curvilinear array transducer (Philips, Netherland) on a frozen monitor image with electronic calipers.

### Definitions

The BMI classification used in this study observed the World Health Organization criteria for Asian populations [35]. Overweight and obesity (OWO) was defined as a BMI ≥ 24.0 kg/m^2^, and normal weight as a BMI of 18.5–23.9 kg/m^2^. Early FGR was defined as first-trimester gestational age-adjusted CRL in the lowest 20th centile of the population [28]. Gestational age was based on date of last menstrual period (LMP) reported by the patient. CRL values were transformed to standard deviation scores (SDS) standardized to gestational age of pregnancy determined according to LMP, which display as CRL (SDS).

### Statistical analysis

The primary outcome of interest was early FGR defined as CRL < 20th centile in the first trimester of pregnancy. The serum 25(OH)D concentrations and BMI were used as both, continuous and categorical variables in the statistical analyses. Descriptive statistics were produced for all study variables. Continuous variables were presented as mean and standard deviation (SD) or mean and 95% confidence interval. Categorical data were presented as frequency and percentage.

We used ordinary least squares regression models to estimate the relationship between BMI and VitD with CRL (SDS). *P* value < 0.05 was considered statistically significant.

We studied the combined effects of maternal serum 25(OH)D concentrations and prepregnancy BMI on CRL using generalized linear regression model. A heat map was constructed to display CRL differences according to combinations of prepregnancy BMI and 25(OH)D concentration, where red color indicates a higher CRL and blue color indicates a lower CRL. Furthermore, we tested the interaction of CRL and BMI on birthweight. In combined effect analysis, *P* value for interaction < 0.15 was considered statistically significant [36].

We also estimated the risk of newborn adverse outcomes according to categories of VitD concentrations using logistic regression models. No significant differences in descriptive characteristics were found between the original and imputed data sets using ANOVA or chi-square analysis. All statistical analyses were performed using R v3.04 (package MASS, rms and visreg) or SPSS Statistics for Windows v20.0 (IBM Corp., Armonk, NY) with *P* value < 0.05 considered to be statistically significant.

## Results

### Demographic and clinical characteristics

The demographic and clinical characteristics of the 15,651 mother-infant dyads included in the study are presented in Table 1. The average maternal age was 30.4 years, and the median prepregnancy BMI was 20.98 kg/m^2^. The majority of women were normal weight, 13.3% were underweight and 11.2% were overweight and obese. The majority (76.04%) of women were nulliparous. The average gestational age at delivery was 38.9 weeks and 42.43% of women were delivered by C-section. During pregnancy, 10.31%, 2.42%, and 0.71% of the women were diagnosed with gestational diabetes mellitus, pregnancy-induced hypertension, and intrahepatic cholestasis of pregnancy, respectively. The seasonal distribution of VitD testing was almost equal between Winter, Fall and Spring season, with Summer having the lowest percentage of testing, of 23.44%. Regarding newborn characteristics at birth, the incidence rates of preterm birth (< 37 weeks), SGA, and low birthweight (< 2500 g) were 5.27%, 3.64%, and 2.91%, respectively. Comparing maternal characteristics between VitD groups, we found that the number of women with raised prepregnancy BMI was significantly higher in VitD INSUFF/DEF group than in SUFF group (20.71 vs 21.09; *P* < 0.001). The VitD concentration distribution in this study population is shown in Supplemental Fig. 1.

**Table 1:** Clinical characteristics of the study population (*n* = 15,651)

	Total sample	Vitamin D SUFF^a^	Vitamin D INSUFF/DEF^b^	*P*
Maternal characteristics				
Age, mean (SD), y	30.36 (3.68)	30.62 (3.69)	30.24(3.72)	< 0.0001
Prepregnancy BMI, mean (SD), kg/m^2^	21.98 (2.63)	20.71 (2.58)	21.09(2.73)	< 0.0001
BMI groups				< 0.0001
BMI, 18.5–23.9 kg/m^2^, *n* (%)	11,827 (75.56)	3852 (75.90)	7975(75.41)	
BMI, < 18.5 kg/m^2^, *n* (%)	2074 (13.26)	774 (15.25)	1300 (12.29)	
BMI, ≥ 24 kg/m^2^, *n* (%)	1750 (11.18)	449 (8.85)	1301 (12.30)	
Gestational age, mean (SD), weeks	38.9 (1.39)	38.8 (1.32)	38.9 (1.43)	< 0.0001
Parity, *n* (%)				< 0.0001
Nulliparous	11,902 (76.04)	3654 (72.00)	8248 (77.98)	
Multiparous	3749 (23.95)	1421 (28.00)	2328 (21.02)	
C-section, *n* (%)	6641 (42.43)	2163 (42.62)	4478 (42.34)	0.74
GDM, *n* (%)	1613 (10.31)	559 (11.01)	1054 (9.96)	0.05
PIH, *n* (%)	379 (2.42)	109 (2.15)	270 (2.55)	0.12
ICP, *n* (%)	111 (0.71)	33 (0.65)	78 (0.74)	0.54
Season at VD testing				< 0.0001
Spring (March–May)	3986 (25.47)	1199 (23.63)	2787 (2635)	
Summer (June–August)	3669 (23.44)	1492 (29.39)	2177 (20.59)	
Autumn (September–November)	3959 (25.30)	1403 (27.65)	2556 (24.17)	
Winter (December–February)	4037 (25.79)	981 (19.33)	3056 (28.89)	
Neonatal characteristics				
Male sex, *n* (%)	8096 (51.73)	2675 (52.71)	5421 (51.26)	0.09
Birthweight, mean (SD), g	3341.7 (438.10)	3335.7 (433.35)	3344.5 (440.28)	0.24
Preterm birth (< 37 wk), *n* (%)	825 (5.27)	272 (5.35)	553 (5.23)	0.73
SGA, *n* (%)	570 (3.64)	172 (3.39)	398 (3.76)	0.24
Low birthweight (< 2500 g), *n* (%)	456 (2.91)	144 (2.84)	312 (2.95)	0.69
LGA, *n* (%)	2166 (13.84)	667 (13.14)	1499 (14.17)	0.08
High birthweight (≥ 4000 g), *n* (%)	930 (5.94)	282 (5.55)	648 (6.13)	0.16

*BMI* body mass index (calculated as weight in kilograms divided by height in meters squared), *GDM* gestational diabetes mellitus, *ICP* intrahepatic cholestasis of pregnancy, *LGA* large for gestational age, *PIH* pregnancy-induced hypertension, *SGA* small for gestational age

^a^SUFF (sufficient), 25(OH)D in serum ≥ 50.00 nmol/L

^b^INSUFF/DEF (insufficient or deficient), 25(OH)D in serum < 50.00 nmol/L

### Prepregnancy OWO, maternal VitD concentrations and CRL in first trimester

**Fig. 2:**
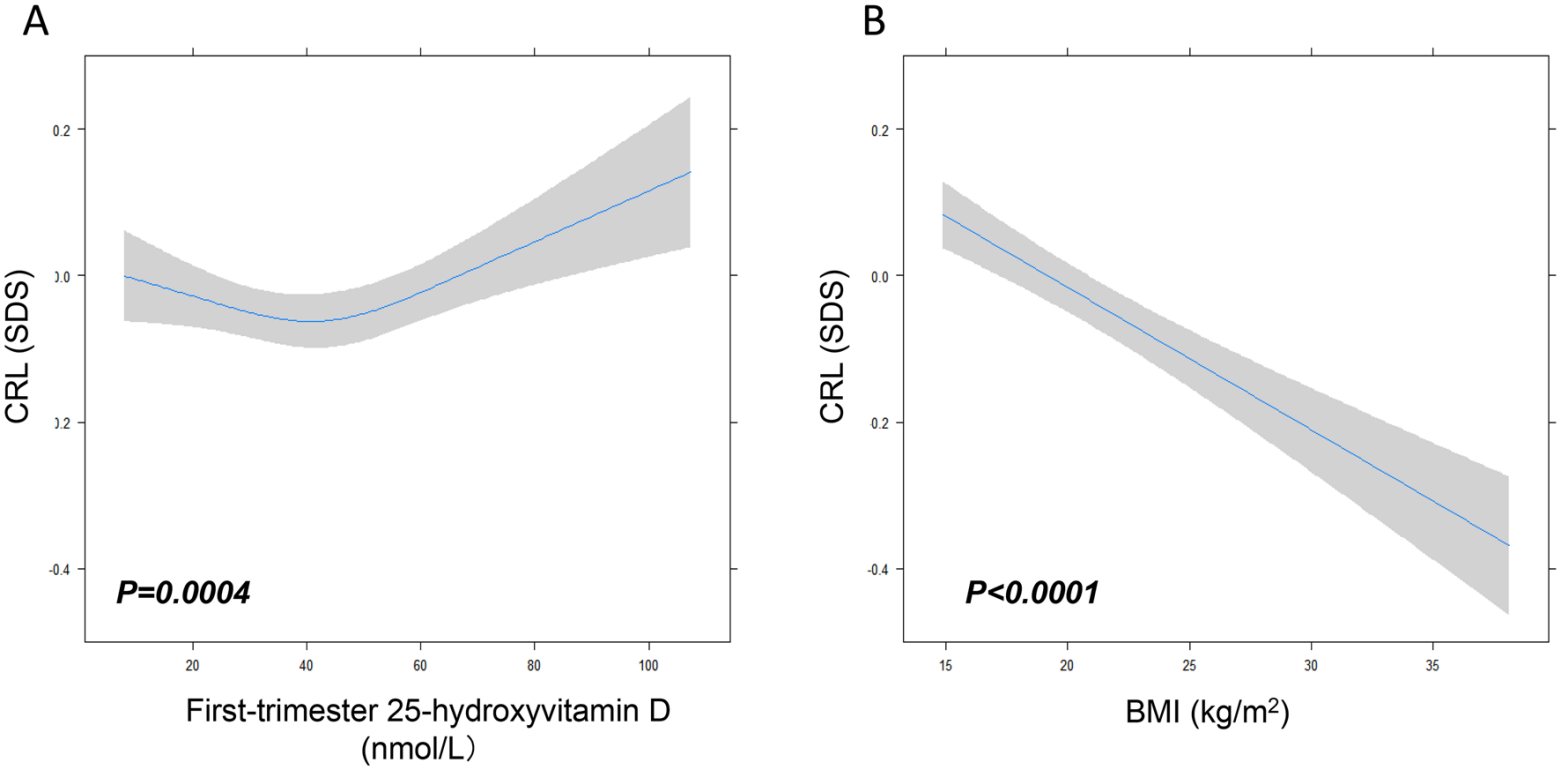
The association of maternal BMI and Vitamin D status with CRL in early pregnancy. Plots show the linear regression models for first-trimester vitamin D (**A**), prepregnancy BMI (**B**) and CRL as predicted mean (line) with CI (gray area) in the full range of CRL. *n* = 15,651, *P* values are based on the ordinal linear regression model with three knots of restricted cubic spline. Analyses were adjusted for maternal age, BMI, parity, season at vitamin D testing, fetal sex. *BMI* body mass index, *CRL* crown-rump length

As shown in Fig. 2A, there was an association between maternal first trimester VitD concentrations, prepregnancy BMI and fetal size. Among women with SUFF VitD levels, there was a steep rise in CRL with increasing maternal 25(OH)D concentration (*P* = 0.004). For women with INSUFF/DEF levels of VitD, the CRL showed a decreasing trend with the increase in 25(OH)D levels, although the trend was a slow descent in CRL.

As shown in Fig. 2B, there was an inverse association between maternal prepregnancy BMI and the first trimester CRL. A higher BMI was associated with a lower CRL, with an approximately 0.4 SD lower CRL across the full range of BMI (*P* < 0.0001).

As shown in Table 2, CRL in the VitD INSUFF/DEF group was lower by 0.05 SD, that is, 0.325 mm, compared with the VitD SUFF group (*P* = 0.005). The fetuses in the maternal VitD INSUFF/DEF group were associated with a 13% higher risk for early FGR (95% CI 1.04–1.24, *P* = 0.004) compared with those in the SUFF group.

**Table 2:** The association of maternal vitamin D concentrations and pregregnancy BMI categories with CRL in early pregnancy

Maternal characteristic	Effect size for CRL standard deviation score (SDS),1 SDS = 6.5 mm			Restricted fetal growth (CRL < 20th percentile)		
	Mean (SE)	Adjusted mean differences (95% CI)	*P*	*n* (%)	Adjusted OR (95% CI)	*P*
Vitamin D binary						
SUFF^a^	0.06 (0.02)	0 [Reference]	1 [Reference]	928 (18.3)	0 [Reference]	1 [Reference]
INSUFF/DEF^b^	0.01 (0.01)	− 0.05 (− 0.08, − 0.02)	0.005	2202 (20.8)	1.13 (1.04, 1.24)	0.004
Vitamin D, nmol/L						
p5(< 20.0)	− 0.03 (0.04)	− 0.08 (− 0.18, 0.02)	0.12	164 (21.5)	1.31 (1.01, 1.71)	0.04
p5-p25(20.0 -30.5)	− 0.004 (0.02)	− 0.07 (− 0.15, 0.003)	0.06	627 (20.0)	1.23 (1.00, 1.52)	0.06
p25-p50(30.6 -41.6)	− 0.03 (0.02)	− 0.11 (− 0.19, − 0.04)	0.004	826 (21.1)	1.35 (1.09, 1.66)	0.005
p50-p75(41.7 -53.5)	− 0.02 (0.02)	− 0.10 (− 0.18, − 0.03)	0.009	810 (20.6)	1.31 (1.07, 1.62)	0.01
p75-p95(53.6 -70.4)	0.03 (0.02)	− 0.07 (− 0.15, 0.01)	0.08	578 (18.5)	1.16 (0.94, 1.44)	0.16
p95-p100(≥ 70.5)	0.12 (0.03)	0 [Reference]		125 (15.8)	0 [Reference]	
*P* for trend	0.002			0.003		
BMI, kg/m^2^						
Underweight (< 18.5)	0.08 (0.02)	0.04 (− 0.01, 0.08)	0.11	402 (19.4)	0.95 (0.84, 1.07)	0.37
Normal weight (18.5–23.9)	0.05 (0.01)	0 [Reference]	1 [Reference]	2273 (19.2)	0 [Reference]	1 [Reference]
OWO (≥ 24)	− 0.15 (0.02)	− 0.20 (− 0.25, − 0.15)	< 0.0001	455 (26.0)	1.58 (1.40, 1.78)	< 0.0001
*P* for trend	< 0.0001			< 0.0001		

*n* = 15,651. General linear regression models provide regression coefficients (*β*) for the standard deviation score (SDS) in CRL. Logistic regression models provide OR (95% CI) for the risk in restricted fetal growth (CRL < 20th centile). Analyses were adjusted for maternal age, parity, season at vitamin D testing, fetal sex

*BMI* body mass index, *CRL* crown-rump length, *OWO* overweight or obese

^a^SUFF (sufficient), 25(OH)D in serum ≥ 50.00 nmol/L

^b^INSUFF/DEF (insufficient or deficient), 25(OH)D in serum < 50.00 nmol/L

Using top 5% (95–100th percentile (p95-p100), ≥ 70.5 nmol/L) 25(OH)D concentration as a reference, we found that fetuses of mothers with p50-p75, p25-p50 25(OH)D concentrations had a 0.10, 0.11 SDS decrease (*P* = 0.009, 0.004) in CRL and a 1.31-fold (95% CI 1.07–1.62, *P* = 0.01), and 1.35-fold (95% CI 1.09–1.66, *P* = 0.005) increased risk for FGR, respectively. Fetus of mothers within the bottom 5% (< p5, < 20.0 nmol/L) 25(OH)D levels had a 1.31-fold (95% CI = 1.47–2.29, *P* = 0.04) increased risk for FGR, compared to the fetuses of mothers within top 5th percentile of the 25(OH)D levels.

The CRL of the fetuses in the prepregnancy OWO group was significantly lower, by 0.15 SD, that is, 1.30 mm, when compared to the fetuses of women with normal prepregnancy weight group (*P* < 0.0001). The fetuses in the maternal OWO group were associated with a 58% higher risk for early FGR (95% CI 1.40–1.78, *P* < 0.001) compared with those in the normal-weight group.

**Fig. 3:**
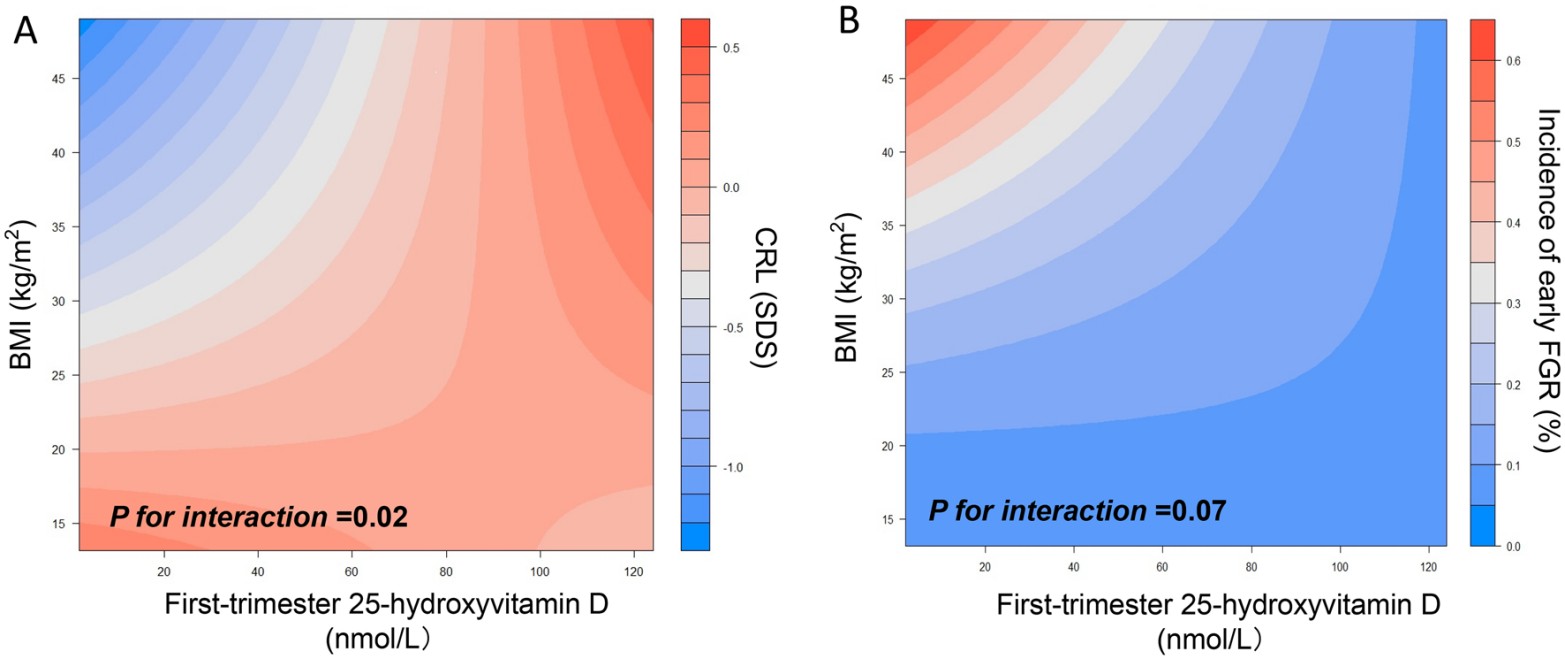
Combined effect of maternal vitamin D concentrations and pregregnancy BMI on early fetal growth. Panel **A** displays a heat map for the association of first trimester CRL (red color indicates higher CRL, blue color indicates lower CRL) according to the interaction of vitamin D-BMI. Panel **B** displays a heat map for the association of incidence of FGR (red color indicates higher incidence; blue color indicates lower incidence) according to the interaction of vitamin D-BMI. *n* = 15,651, *P* values are based on the ordinal linear regression model by adding a product interaction term to the model (First trimester Vitamin D × BMI)

Taken together, there was a significant combined effect of maternal VitD concentrations and prepregnancy OWO on first trimester CRL, which is shown in the heat map from Fig. 3A. Combined effects of VitD and BMI determined more pronounced changes in the values of CRL measurements (*P* for interaction = 0.02) than VitD or BMI alone. A low 25(OH)D concentration in early pregnancy in OWO women was associated with an up to 1.0 SD lower CRL, whereas 25(OH)D concentration above 120 nmol/L in early pregnancy was associated with a 0.5 SD higher CRL despite a high BMI. Additionally, we found that the combination of increased 25(OH)D levels and high maternal BMI had significant effects on CRL of both female (*P* for interaction = 0.03, Supplemental Fig. 2A) and male fetuses (*P* for interaction = 0.10, Supplemental Fig. 2B).

Meanwhile, the incidence of early FGR was significantly changed with the combined effects of VitD and BMI (Fig. 3B, *P* for interaction = 0.07). Increase in 25(OH)D concentration in OWO mothers was accompanied by a decrease in the incidence of early FGR. For instance, 25(OH)D concentration above 120 nmol/L in early pregnancy had an incidence of FGR as low as 0.1% despite a high BMI. We also found that higher BMI was associated with lower maternal 25(OH)D concentration (*P* < 0.0001; Supplemental Fig. 3).

## Discussion

To our knowledge, this is the first study to evaluate both, the individual and the combined effects of maternal early pregnancy serum VitD concentrations and prepregnancy BMI on early fetal growth. Our results revealed a strong association between early pregnancy VitD concentrations and prepregnancy BMI with first trimester CRL. Our findings provide evidence to support that maternal VitD status may play an important role in early FGR, association that is mediated by maternal body habitus.

To date, evidence of the relationship between maternal BMI and early fetal growth is scarce and controversial. Previous studies suggested that low first-trimester CRL is associated with increased risks of adverse birth outcomes, including preterm birth, SGA, low birthweight, as well as long-term programming effects on childhood and adulthood health [28, 37–40]. A study by Sarris et al. reported that prepregnancy BMI does not influence first trimester fetal growth [41]. A reason for such findings may reside in the heterogenous population of the study, that included women with prepregnancy diabetes and thyroid disease, both comorbidities which have been shown to affect early fetal growth [29–31].

There is, however, mounting evidence on the essential role of VitD on pregnancy health. VitD plays a significant role in early embryonic and fetal growth. Placental 25(OH)D status and VitD receptor (VDR) mRNA expression levels are significantly decreased in pregnancies affected by idiopathic FGR [42]. The observations from this study, where we also report that low circulating levels of VitD influence early embryonic growth, especially in OWO mothers, align with the previous studies. In agreement with our findings, other studies have also shown that VitD deficiency is more common in OWO women [16, 43]. A recent systematic review shows that the prevalence of VitD deficiency is 35% higher in obese and 24% higher in the overweight subjects compared to the eutrophic group [16]. The relationship between VitD deficiency and obesity is mediated through several mechanisms, including the decreased bioavailability of VitD_3_ [15] and increased adipogenesis dependent on the inhibition of peroxisome proliferator-activated receptor gamma (PPARγ) and CCAAT/enhancer binding protein alpha (C/EBPα) modulated by 1,25-hydroxyvitamin D through VDR [44].

However, the interaction effects between maternal VitD concentrations and prepregnancy BMI with FGR are still not clear. In the present study, we observed a combined effect of maternal VitD levels and OWO status on the first trimester CRL, although we could not demonstrate an additive effect. We report that, in OWO mothers, sufficient maternal VitD levels associate with a decreased risk for FGR in the first trimester. Based on available evidence, we hypothesize that the physiologic mechanisms of this association my involve adiponectin effects on early fetal growth. It has been reported that obesity in pregnancy is associated with downregulation of adiponectin expression in the maternal adipose tissue and hypoadiponectinemia, which in turn will stimulate pregnancy-induced insulin resistance [45], with potential effects on fetal growth. Previous animal and human studies have indicated that VitD levels are directly associated with adiponectin, and the association varies across BMI categories, stronger with increasing BMI [46, 47]. Furthermore, reduced adiponectin levels in mothers was associated with intrauterine growth restriction, association that is influenced by the percentage of adipose tissue and insulin resistance [48, 49]. Taken together, it is conceivable that adiponectin may contribute to the interaction effects between maternal VitD concentrations and prepregnancy BMI with FGR. Further research is warranted to study more in depth these associations and their underlying physiological mechanisms.

The main strength of our study is represented by its large and homogeneous population. We have excluded mothers with prepregnancy diabetes and thyroid disease, which are known to contribute to adverse fetal outcomes and influence fetal growth. We started our study with a larger cohort that allowed us safe exclusion of the maternal–fetal dyads that had incomplete data. The remaining cohort included in the analysis was sizable and representative for our population. However, our study has several limitations that should be acknowledged. This study was monocentric, including only the Chinese women from the Shanghai area. Furthermore, maternal prepregnancy BMI was primarily based on self-reported height and weight, thus it may have been subject to reporting bias. The proportion of obese women in our population was relatively small compared to the reported in western populations, therefore, we merged overweight and obesity in one category for meaningful comparisons. We variation potentially introduced by season of vitD evaluation was not considered in this study. Also, we did not assess the levels of parathormone (PTH) in relation with VitD levels and early fetal growth [50]. Future studies are warranted to examine the individual and combined effects of PTH and VitD on fetal growth.

In conclusion, this study provides insight into the complex relationship between maternal characteristics and fetal growth early in pregnancy. Sufficient VitD in the first trimester has protective effects on early fetal growth, predominantly in OWO women. Identifying the factors that influence first trimester fetal growth would facilitate guidance for prenatal care and development of interventions from early pregnancy or pre-conception period to improve pregnancy outcomes.

## Supplementary Information

Below is the link to the electronic supplementary material.

Supplementary file1 (PPTX 2451 KB)

## Data Availability

The datasets used or analyzed during this study are available from the corresponding author on reasonable request.

## Abbreviations

FGR: Fetal growth restriction
BMI: Body mass index
OWO: Obese or overweight
RCTs: Randomized controlled trials
LMP: Last menstrual period
25(OH)D: 25-Hydroxyvitamin D
CRL: Crown-rump length
VitD: Vitamin D
INSUFF/DEF: Insufficient or deficient
SUFF: Sufficient
PTH: Parathormon
