# Supplementary material for: Relationship of maternal obesity and vitamin D concentrations with fetal growth in early pregnancy
Source: Eur J Nutr. 2021 Oct 17;61(2):915–24. doi: 10.1007/s00394-021-02695-w (PMC8854300; doi:10.1007/s00394-021-02695-w)
Supplement: Supplementary file 1 — Supplementary file1 (PPTX 2451 KB) [file 394_2021_2695_MOESM1_ESM.pptx]

## Slide 1
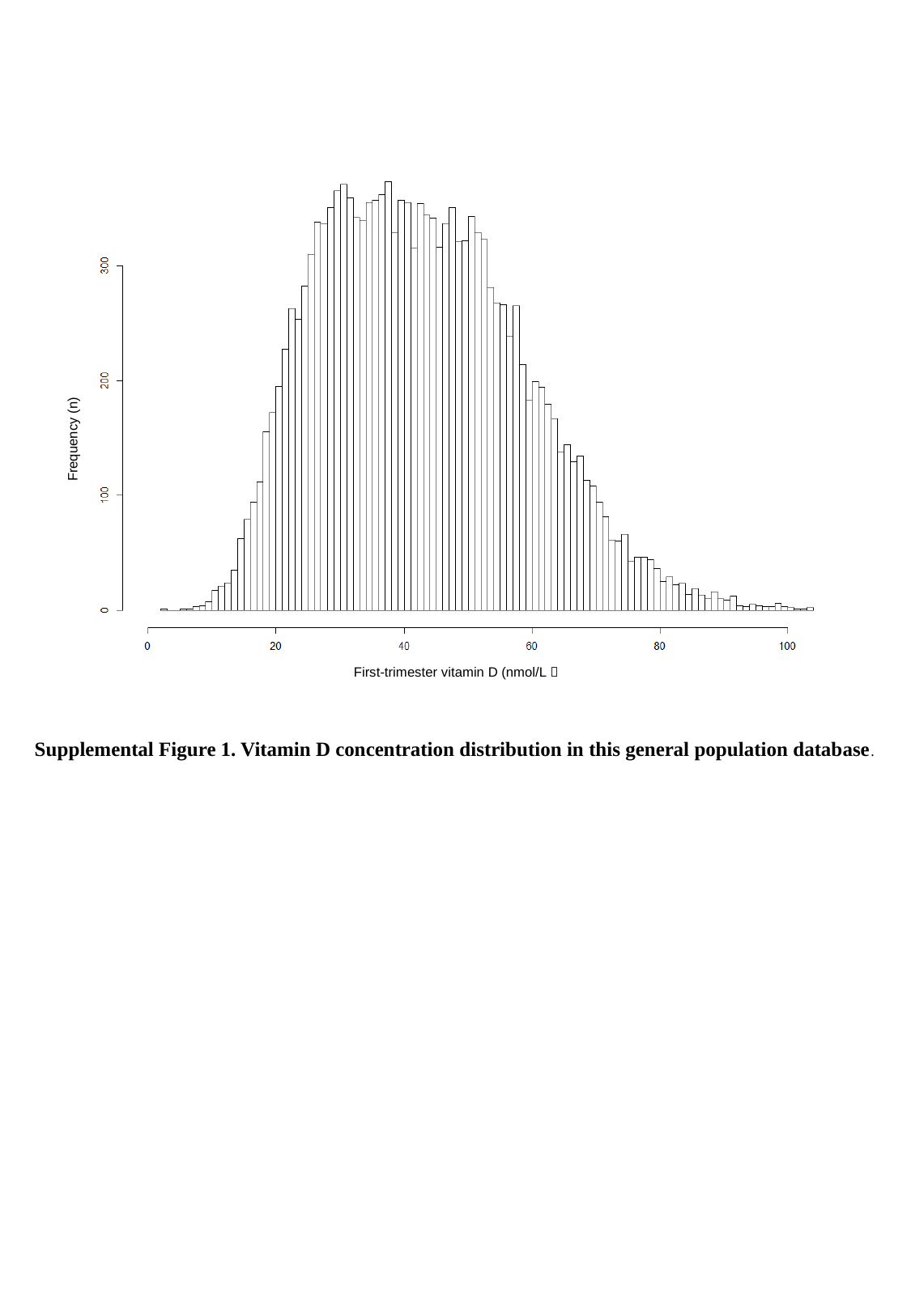

Frequency (n)
First-trimester vitamin D (nmol/L）
Supplemental Figure 1. Vitamin D concentration distribution in this general population database.

## Slide 2
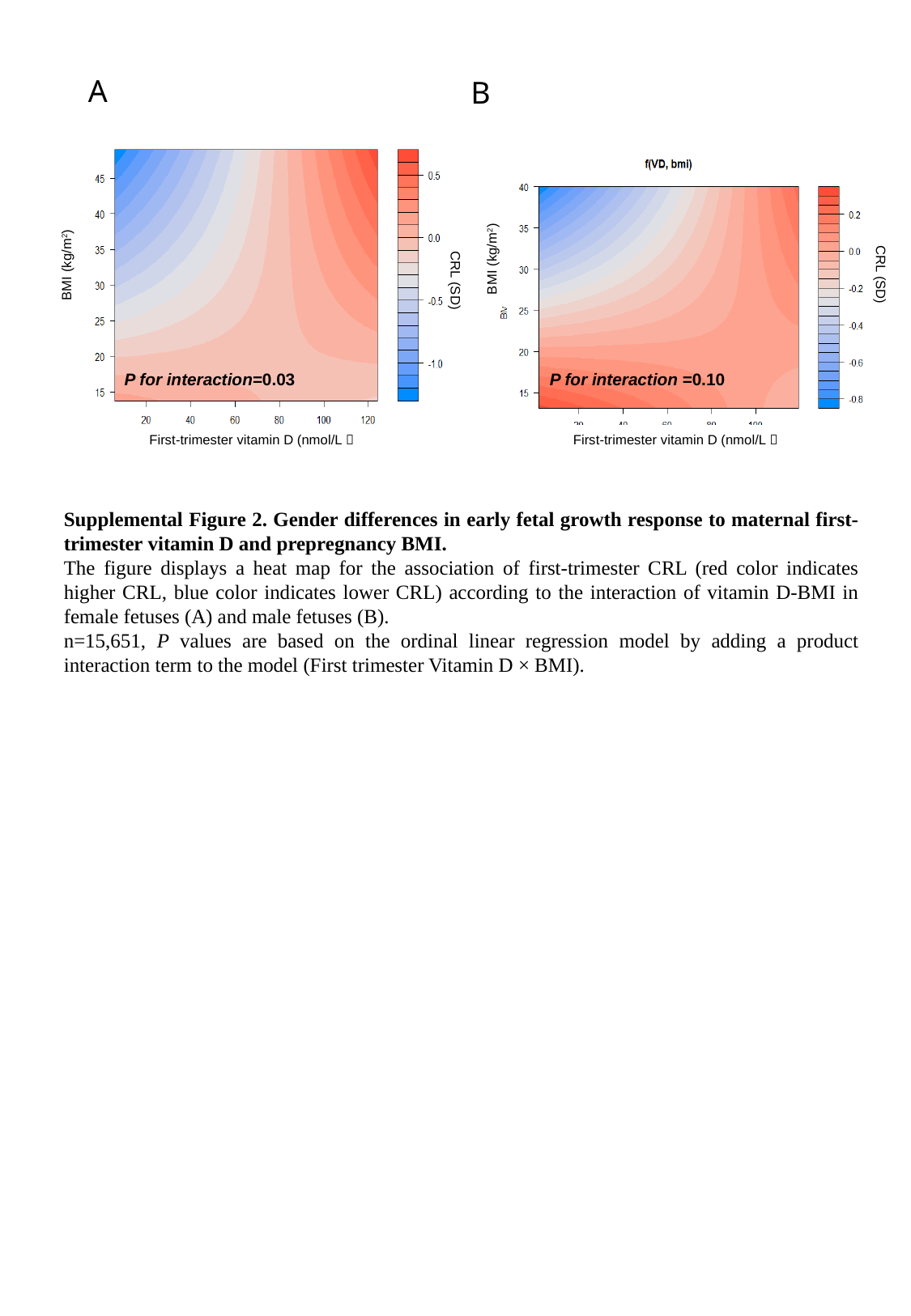

A
B
BMI (kg/m2)
CRL (SD)
P for interaction =0.10
First-trimester vitamin D (nmol/L）
BMI (kg/m2)
CRL (SD)
P for interaction=0.03
First-trimester vitamin D (nmol/L）
Supplemental Figure 2. Gender differences in early fetal growth response to maternal first-trimester vitamin D and prepregnancy BMI.
The figure displays a heat map for the association of first-trimester CRL (red color indicates higher CRL, blue color indicates lower CRL) according to the interaction of vitamin D-BMI in female fetuses (A) and male fetuses (B).
n=15,651, P values are based on the ordinal linear regression model by adding a product interaction term to the model (First trimester Vitamin D × BMI).

## Slide 3
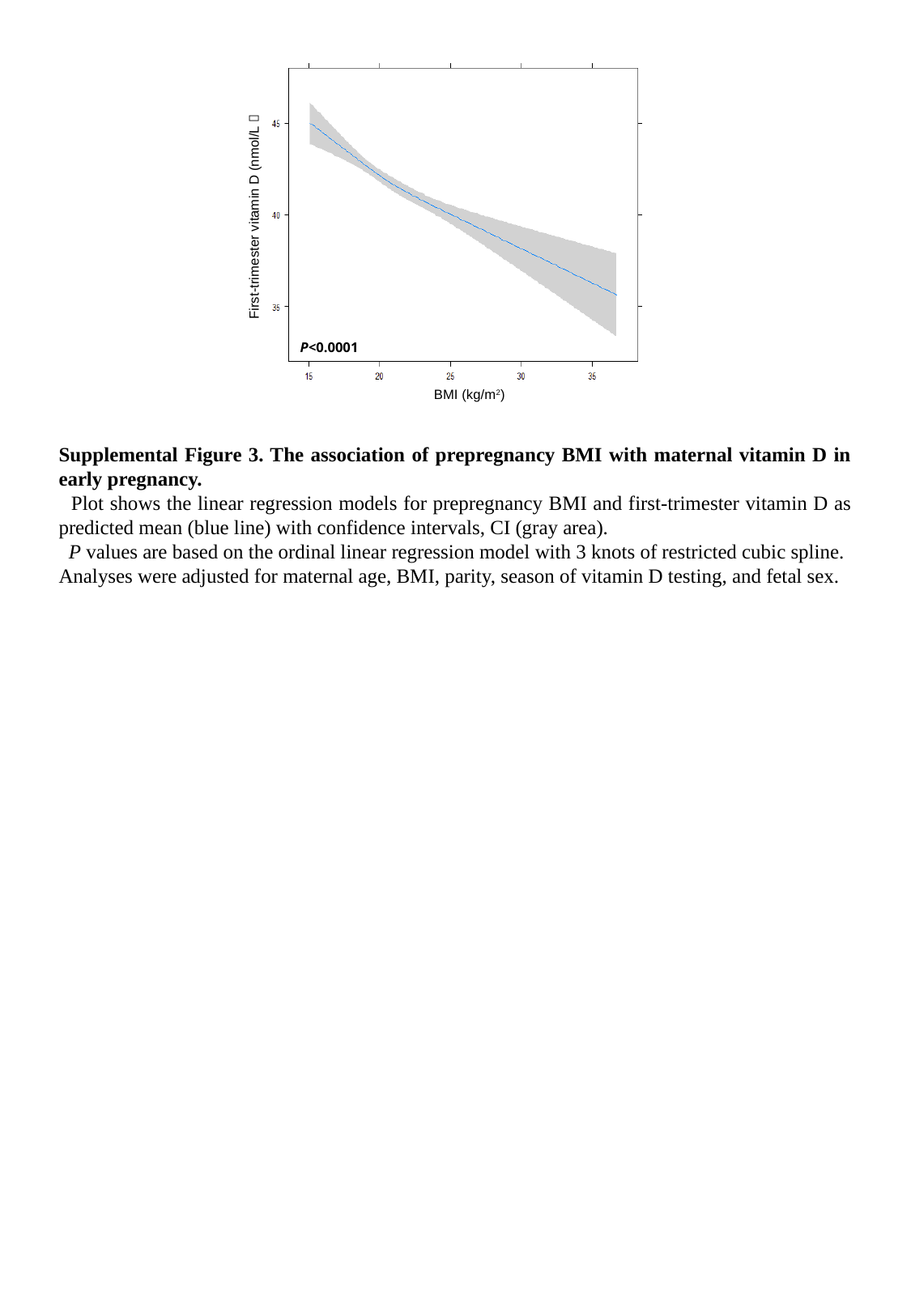

First-trimester vitamin D (nmol/L）
BMI (kg/m2)
P<0.0001
Supplemental Figure 3. The association of prepregnancy BMI with maternal vitamin D in early pregnancy.
 Plot shows the linear regression models for prepregnancy BMI and first-trimester vitamin D as predicted mean (blue line) with confidence intervals, CI (gray area).
 P values are based on the ordinal linear regression model with 3 knots of restricted cubic spline.
Analyses were adjusted for maternal age, BMI, parity, season of vitamin D testing, and fetal sex.
